# Vegetation type modulates negative air ion generation in urban green spaces: the critical role of suburban forests in air quality enhancement

**DOI:** 10.3389/fpubh.2025.1597966

**Published:** 2025-06-17

**Authors:** Yaowen Xu, Jiejie Jiao

**Affiliations:** ^1^Zhejiang Academy of Forestry, Hangzhou, China; ^2^Zhejiang Hangzhou Urban Ecosystem Research Station, Hangzhou, China

**Keywords:** negative air ion, relative humidity, urban green space, forest, city, air quality

## Abstract

Negative air ions (NAIs) are recognized as critical indicators of air quality and environmental well-being, with urban green spaces serving as vital sources of these beneficial ions. However, the spatiotemporal dynamics of NAIs across heterogeneous urban green infrastructure and their environmental determinants remain understudied. In this study, we examined the spatial and temporal distribution of negative air ions concentrations within various urban green spaces, specifically suburban forests, parks, roadside greenery, and community green spaces. A stratified sampling design was implemented across 240 georeferenced sites. At each site, NAIs concentrations were quantified using a three-phase measurement protocol:morning (8:00–9:30), midday (12:30–14:00) and evening (17:00–18:30). Field campaigns were conducted during the summer months (June to August) of 2024. Suburban forest areas showed significantly (*p* < 0.05) elevated NAIs levels compared to park, roadside greenery, and community green spaces, while roadside greenery displayed the lowest NAIs levels. We found that environmental factors, including relative humidity, temperature, air pressure, and particulate matter (PM_10_) concentration, significantly influence NAIs concentrations. Notably, relative humidity and temperature demonstrated a positive impact on NAIs levels, whereas air pressure and PM_10_ concentration showed a negative influence. These findings suggest that vegetation type, microclimatic conditions, and air quality have a crucial role in regulating NAIs generation and distribution. The study provides valuable insights for urban green space planning and management to enhance air quality and promote public health through optimized green infrastructure design.

## 1 Introduction

Negative air ions (NAIs) are gaseous molecules or atomic clusters carrying net negative charges, predominantly generated through ionizing processes including cosmic/solar radiation, Lenard effect from water cascade in natural landscapes (e.g., waterfalls, rainstorms), and bioelectrochemical reactions during plant photosynthetic activities (1). These airborne anions exhibit size-dependent mobility, with smaller clusters demonstrating higher biological activity due to enhanced pulmonary deposition efficiency (2). Epidemiological evidence from longitudinal cohort studies has established NAIs’ dose-dependent associations with improved cardiopulmonary function, upregulated cell activity, and attenuated cortisol levels in saliva biomarkers (2). Urban green spaces, as critical components of urban ecosystems, play a vital role in generating and maintaining high concentrations of NAIs, contributing to the overall well-being of urban residents (3). However, the spatial and temporal dynamics of NAIs concentrations in urban green spaces and their relationship with environmental factors remain poorly understood, particularly in rapidly urbanizing regions like China.

Urbanization has led to significant environmental challenges, including air pollution, heat island effects, and reduced green spaces, all of which negatively impact air quality and human health (4–6). NAIs, as natural air purifiers, can neutralize harmful airborne particles, such as particulate matter, and improve air quality. Higher NAIs concentrations are associated with lower levels of air pollution and better respiratory health outcomes (2, 7). Therefore, understanding the distribution and dynamics of NAIs in urban green spaces is essential for designing healthier urban environments.

Urban green spaces, including parks, gardens, and street trees, are significant sources of NAIs due to the presence of vegetation and water bodies (5, 6). Vegetation, particularly trees and shrubs, enhances NAIs production through processes such as photosynthesis and transpiration (8). Water bodies, such as lakes and fountains, also contribute to NAIs generation through the Lenard effect, where water droplets break apart and release negative ions (9). However, the capacity of different types of green spaces to generate NAIs varies depending on various factors, including plant species, density, and microclimatic conditions.

NAIs concentrations exhibit significant temporal variations, influenced by diurnal and seasonal changes in environmental conditions. For example, NAIs levels are typically higher during the early morning and late afternoon, coinciding with periods of increased photosynthetic activity and lower temperatures (10). Understanding these temporal patterns is crucial for optimizing urban green spaces and maximizing their health benefits. Several environmental factors influence NAIs concentrations in urban green spaces, including temperature, humidity, wind speed, and air pollution levels (11, 12). Higher temperatures and lower humidity levels are associated with lower NAIs concentrations, as these conditions reduce the efficiency of NAIs generation processes (13). Wind speed can also affect NAIs distribution: moderate winds enhance dispersion, while strong winds may reduce overall concentrations. Moreover, air pollution, particularly high levels of particulate matter, can neutralize NAIs and lower their concentrations (14, 15).

Known for its extensive green spaces, notably the West Lake Scenic Area, Hangzhou has prioritized integrating green infrastructure into urban planning. However, the city faces challenges related to air pollution and urban heat islands, making it a representative case for studying the interplay between NAIs and environmental factors in urban settings. Thus, in this study, we aimed to: (1) investigate the temporal dynamics of NAIs concentrations in different urban green spaces in Hangzhou; (2) analyze the relationship between NAIs concentrations and key environmental factors, such as temperature, relative humidity, wind speed, and air pollution levels; and (3) provide practical recommendations for urban planning and green space management to enhance NAIs generation and improve air quality. The results of this study will deepen our understanding of the temporal variation patterns of NAIs concentrations and their influencing factors. They will help to assess and monitor environmental quality more precisely and will provide a basis for pollution control and environmental management.

## 2 Materials and methods

### 2.1 Study area

The study was conducted in Hangzhou, a rapidly urbanizing metropolis in Zhejiang Province, eastern China, renowned for its harmonious blend of ancient cultural heritage and modern ecological urban planning. Situated in a subtropical monsoon climate zone, the city experiences distinct seasonal variations: humid springs (March–May) with frequent drizzle, hot and rainy summers (June–August) influenced by Pacific typhoons, mild and dry autumns (September–November), and cool winters (December–February) with occasional light frost. Detailed climatic parameters recorded during the study period include an annual mean temperature of 17.8°C (peaking at 28.4°C in July and dipping to 4.3°C in January), average relative humidity of 70.3%, substantial annual precipitation of 1,454 mm (predominantly occurring during the May–September plum rain and typhoon seasons), and 1,765 h of sunshine distributed unevenly across seasons. The city’s proactive greening policies, including its “Green City” initiative that has increased urban green coverage to 40.3% since 2000, further enhance the ecological relevance of this study for developing climate-resilient urban planning strategies.

### 2.2 Experimental design

The study focused on four types of urban green spaces: (a) suburban forests, which are natural or semi-natural forest areas in the city outskirts; (b) roadside greenery, consisting of vegetation along urban roads, including street trees and shrubs; (c) park green spaces, which include public parks with a mix of vegetation, water bodies, and recreational facilities; and (d) community green spaces, referring to green areas within residential neighborhoods, such as small gardens and lawns. We selected these green spaces to represent the diversity of urban green infrastructure in Hangzhou and to capture variations in NAIs concentrations due to differences in vegetation type, density, and microclimatic conditions. The study employed a stratified random sampling approach to select monitoring sites within each green space type. We established 240 monitoring sites, evenly distributed across the three green space types, with 60 sites per type. The distance between the monitoring sites was over 200 m, and the sites were selected to ensure the representation of different spatial and environmental conditions, such as proximity to water bodies, traffic density, and vegetation cover.

### 2.3 Observation

The RR-9100 automatic meteorological monitoring system (Yugen, Beijing, China) was used to simultaneously measure monitor air temperature, relative humidity, wind speed, and air pollutant concentrations (CO [Carbon Monoxide], SO_2_ [Sulfur Dioxide], NO_2_ [Nitrogen Dioxide], O_3_ [Ozone], PM_2.5_ [Particulate Matter 2.5], and PM_10_ [Particulate Matter 10]). NAIs concentrations were monitored using an RR-AON1000 sensor (Yugen, Beijing, China) equipped with a detection range of 0 to 1.2 × 10^7^ ions cm^−3^, an ion mobility coefficient of ≥0.4 cm^2^ V^−1^ s^−1^, and a measurement accuracy within ±10%. Concurrently, ambient temperature and relative humidity were recorded with an AV-10TH probe (AVALON Instruments, USA), providing an operational temperature range of −45 to 65°C (accuracy: ±0.2°C) and humidity detection spanning 0–100% RH (accuracy: ±2% RH). To avoid inconsistencies between monitoring instruments, all data were collected using the same type of instruments. The collection frequency was 1 s, with a storage period of 5 min. Measurements were taken at three different time intervals at each monitoring site: morning (8:00–9:30), midday (12:30–14:00) and evening (17:00–18:30). The mean of these periods was calculated as the NAIs levels for each site. The observation period spanned from June to August 2024.

Data preprocessing was adapted from the quality control protocol established by Shi et al. (12), with modifications applied to address sensor-specific anomalies. The implemented screening procedure comprised four sequential steps: (1) Gap removal and anomaly detection: Time series discontinuities arising from instrument maintenance or sensor malfunction were identified and removed through linear interpolation between valid adjacent measurements. (2) Physiological plausibility check: Observations with relative humidity = 0% or NAIs concentration = 0 ions cm^−3^ were flagged as biologically implausible and excluded from analysis (assigned NA). (3) Dynamic threshold filtering: A moving window comparison (*n* = 3) was applied, where data points deviating by more than threefold from preceding and subsequent values within a 5-min interval were classified as transient noise and discarded. (4) Persistence validation: Sequences of ≥6 identical consecutive measurements were interpreted as sensor signal stagnation events and removed to avoid artificial data inflation.

### 2.4 Statistical analyses

One-way analysis of variance was used to determine the effects of urban green space type on NAIs concentrations, air temperature, relative humidity, wind speed, temperature, and air pollutant concentrations (CO, SO_2_, NO_2_, O_3_, PM_2.5_, and PM_10_). Statistical significance was set at *p* < 0.05. Correlation analysis was conducted to determine relationships between NAIs and environmental variables. Random forest analysis was used to assess the relative importance of environmental variables in predicting NAIs. Stepwise regression analysis was used to identify all significant independent variables affecting NAIs. Statistical analyses were conducted using SPSS (version 23.0; IBM, Armonk, NY, USA), and R (version 4.0.2; https://www.r-project.org/).

## 3 Results

### 3.1 Comparison of NAIs concentrations and environmental factors in different urban green spaces

**Figure 1:**
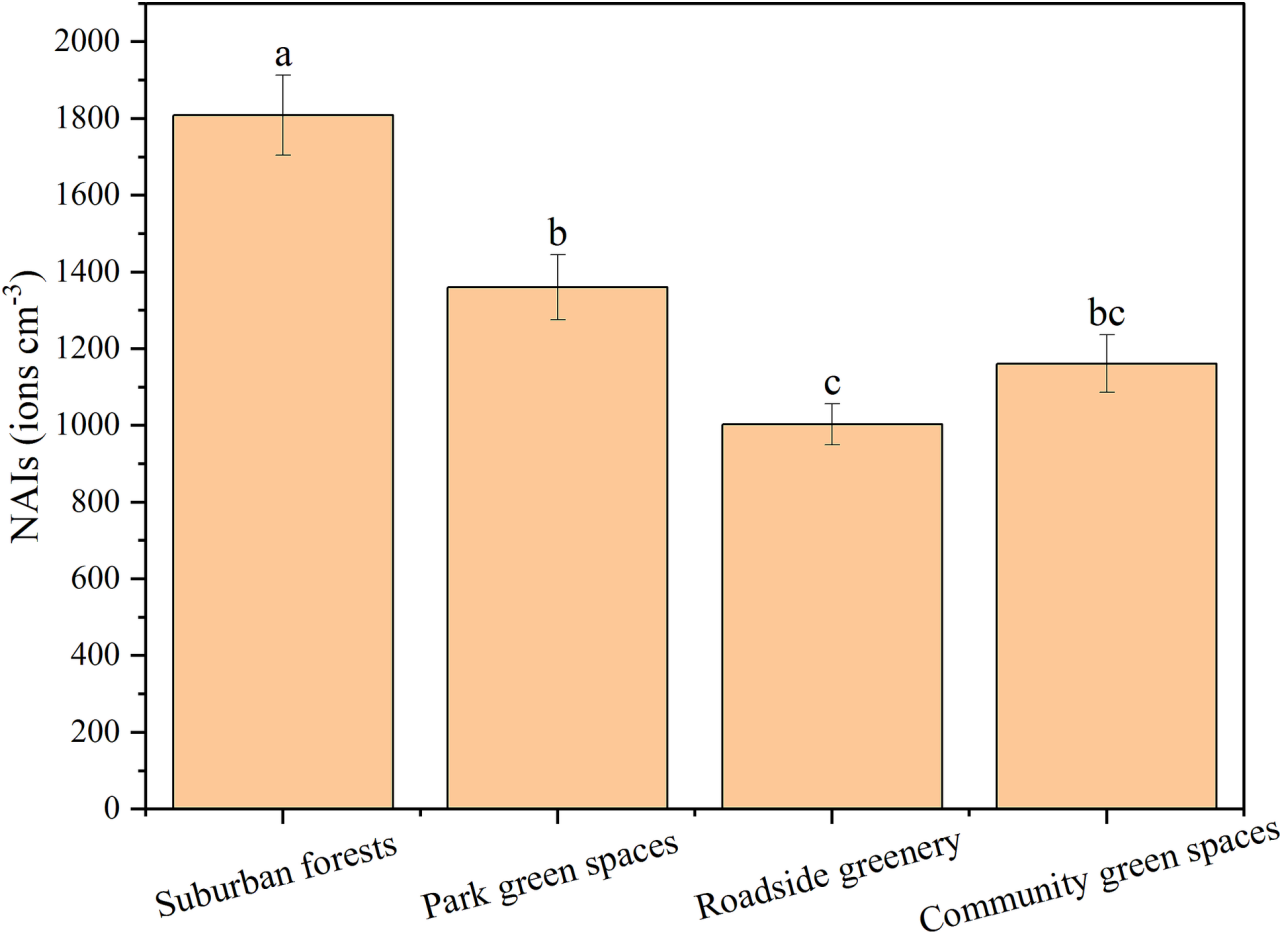
Negative air ion (NAIs) concentrations in four types of urban green spaces. Lowercase letters denote significant differences between green space types (*p* < 0.05).

Across the four green space categories (Figure 1), NAIs concentrations ranged from 1003.35 to 1809.46 ions cm^−3^. The NAIs concentration in suburban forests was significantly higher than in park green spaces, roadside greenery, and community green spaces (*p* < 0.05). Among the four green space types, suburban forests had the highest relative humidity (58.27%). Wind speeds in suburban forests, roadside greenery, and community green spaces were significantly higher than in park green spaces (*p* < 0.05). Community green spaces had the highest temperature among the four types of urban green spaces (*p* < 0.05). However, differences in CO, NO_2_, and O_3_ concentrations among the four urban green spaces were not significant (*p* > 0.05). Suburban forests exhibited the highest SO_2_ concentration (18.35 μg m^−3^) among the four green space categories. The maximum values of PM_2.5_ and PM_10_ appeared in park green spaces and roadside greenery, respectively (Table 1).

**Table 1:** Environmental factors in four types of urban green spaces.

Item	Suburban forests	Park green spaces	Roadside greenery	Community green spaces
Air pressure (hPa)	1003.36 ± 1.05 b	1014.33 ± 0.38 a	1014.81 ± 0.30 a	1014.87 ± 0.42 a
Relative humidity (%)	58.27 ± 1.74 a	54.30 ± 1.63 ab	55.21 ± 1.75 ab	50.59 ± 1.84 b
Wind speed (m s^−1^)	1.01 ± 0.06 a	0.69 ± 0.04 b	0.93 ± 0.06 a	0.91 ± 0.06 a
Temperature (°C)	26.17 ± 0.46 b	27.41 ± 0.37 b	26.67 ± 0.19 b	29.22 ± 1.00 a
CO concentration (μg m^−3^)	0.042 ± 0.00052 a	0.042 ± 0.00054 a	0.042 ± 0.00054 a	0.043 ± 0.00102 a
SO_2_ concentration (μg m^−3^)	18.35 ± 2.59 a	11.18 ± 0.94 b	12.87 ± 1.48 ab	13.32 ± 2.24 ab
NO_2_ concentration (μg m^−3^)	12.15 ± 0.66 a	11.75 ± 0.58 a	11.77 ± 0.51 a	12.10 ± 0.61 a
O_3_ concentration (μg m^−3^)	22.08 ± 1.68 a	18.85 ± 1.22 a	19.70 ± 1.11 a	22.22 ± 1.08 a
PM_2.5_ concentration (μg m^−3^)	10.22 ± 0.72 b	13.42 ± 0.79 a	12.50 ± 0.85 ab	13.20 ± 1.52 ab
PM_10_ concentration (μg m^−3^)	17.18 ± 0.93 b	20.98 ± 1.03 ab	24.52 ± 2.58 a	21.43 ± 1.55 ab

Lowercase letters denote significant differences between green space types (*p* < 0.05).

### 3.2 Effects of environmental factors on NAIs concentration

**Figure 2:**
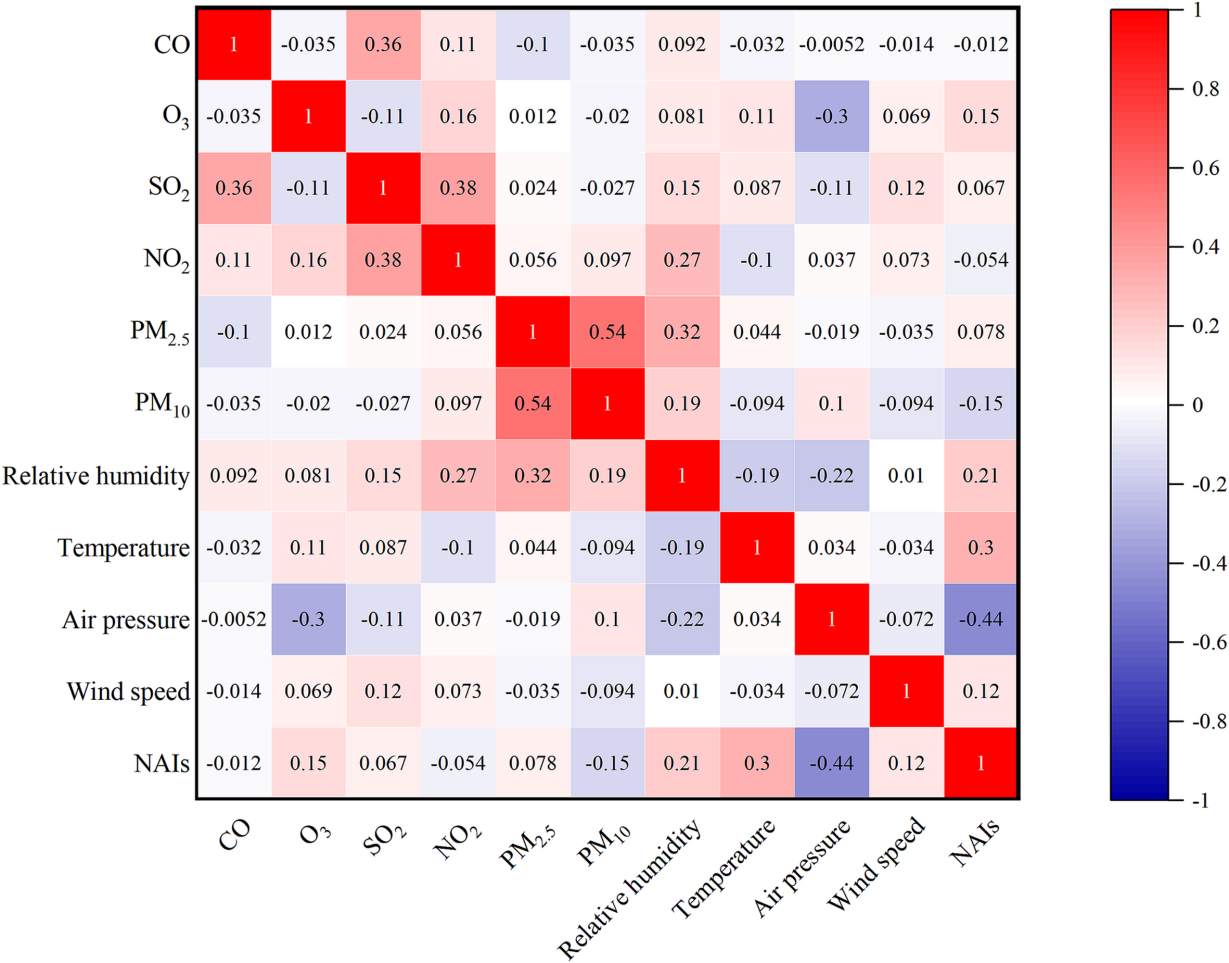
Correlation between negative air ions (NAIs) and environmental factors. Blue and red in the square represent negative and positive correlations, respectively. CO, Carbon Monoxide; SO₂, Sulfur Dioxide; NO₂, Nitrogen Dioxide; O₃, Ozone; PM₂.₅, Particulate Matter 2.5; PM₁₀, Particulate Matter 10.

**Figure 3:**
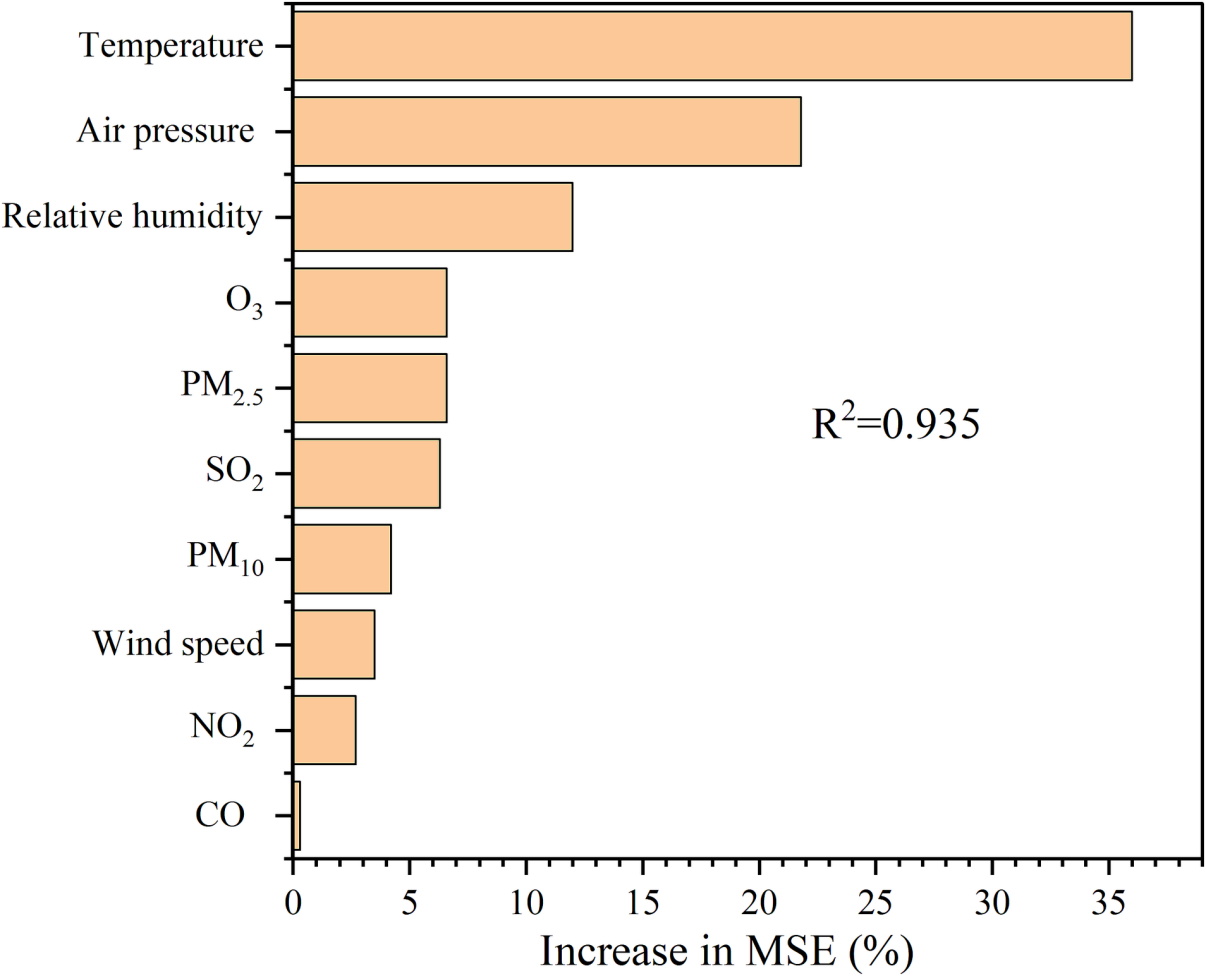
Relative importance of environmental factors using random forest for negative air ions (NAIs) concentration. CO, Carbon Monoxide; SO₂, Sulfur Dioxide; NO₂, Nitrogen Dioxide; O₃, Ozone; PM₂.₅, Particulate Matter 2.5; PM₁₀, Particulate Matter 10.

NAIs concentrations were closely associated with multiple environmental factors (Figure 2). Specifically, NAIs significantly increased with the increase in several environmental variables, including temperature and relative humidity (all *p* < 0.01; Figure 2). Additionally, NAIs exhibited a positive correlation with O_3_ (*p* < 0.05; Figure 2) but were negatively correlated with air pressure and PM_10_ (*p* < 0.01 and *p* < 0.05, respectively; Figure 2). The random forest regression analysis revealed that temperature had the greatest influence on NAIs, contributing 36.1% to the observed variations (Figure 3).

A stepwise regression analysis was performed, treating all environmental factors as independent variables and NAIs as dependent variables. After automatic model selection, four environmental factors were retained: temperature, relative humidity, air pressure, and PM_10_ (Table 2). The model passed the F-test (*F* = 29.91, *p* < 0.01), confirming its validity. Additionally, we tested the multicollinearity of the model and found that all VIF values were below 5, indicating no collinearity problem. The summary analysis indicated that temperature and relative humidity had significant positive effects on NAIs, whereas air pressure and PM_10_ had significant negative effects.

**Table 2:** Stepwise regression analysis of negative air ions (NAIs) and environmental factors. *p < 0.05; **p < 0.01.

Item	Non-normalized coefficient	Normalized coefficient	*t*	*p*	VIF	*R* ^2^	Adjusted *R*^2^	*F*
Constant	40378.16		7.05	0.00**		0.34	0.33	29.91
Temperature	51.22	0.34	6.34	0.00**	1.04			
Relative humidity	10.52	0.21	3.66	0.00**	1.13			
Air pressure	−40.41	−0.40	−7.19	0.00**	1.07			
PM_10_	−6.11	−0.11	−2.08	0.04*	1.06			

## 4 Discussion

### 4.1 Comparison of NAIs concentrations in different urban green spaces

The findings of this study reveal significant variations in NAIs concentrations across different urban green spaces, with suburban forests exhibiting the highest NAIs levels, followed by park green spaces, community green spaces, and roadside greenery. These results align with previous research highlighting the critical role of vegetation type, density, and environmental conditions in influencing NAIs generation and distribution (16, 17). The observed patterns can be attributed to several factors, including differences in vegetation structure, microclimatic conditions, and proximity to pollution sources (18). Suburban forests consistently demonstrated the highest NAIs concentrations among the studied green spaces, which can be explained by the dense vegetation cover, high tree species diversity, and minimal anthropogenic disturbances in these areas. Forests enhance NAIs production through processes such as photosynthesis, transpiration, and the release of volatile organic compounds (19). The presence of water bodies, such as streams or ponds, in suburban forests further contributes to the NAIs generation through the Lenard effect, where water droplet fragmentation releases negative ions (15). The combination of these factors creates an optimal environment for high NAIs concentrations, making suburban forests a critical component of urban green infrastructure for improving air quality and human health. Park green spaces exhibited moderate NAIs concentrations, lower than suburban forests but higher than community green spaces and roadside greenery. Parks typically feature a mix of vegetation, water bodies, and open spaces, which collectively contribute to NAIs generation (9). However, the relatively lower NAIs levels in parks compared to suburban forests may be caused by extensive human activity, which can disturb vegetation and reduce NAIs production (20). Despite this, parks remain important urban green spaces due to their accessibility and recreational value, offering a balance between NAIs benefits and public usability. Roadside greenery, including street trees and shrubs, exhibited the lowest NAIs concentrations among the studied green spaces (21), which can be attributed to several factors, including proximity to traffic-related pollution, limited vegetation density, and harsh microclimatic conditions (22). Vehicle emissions release particulate matter and gaseous pollutants, which can neutralize NAIs and reduce their concentrations (11). The compacted soil and limited root space in roadside greenery can restrict plant growth and reduce their capacity to generate NAIs (11). Despite these challenges, roadside greenery remains essential for mitigating urban heat islands and providing aesthetic benefits. The differential NAIs production across vegetation types likely stems from synergistic interactions between biochemical and biophysical mechanisms. For instance, coniferous species exhibit elevated NAIs generation through two primary pathways: (1) phytoncide emissions that undergo photooxidation to release electrons, and (2) needle-shaped foliage creating localized electrostatic fields via triboelectric charging during wind interactions, enhancing aerosol deposition and subsequent charge liberation. Broadleaf species conversely demonstrate stronger humidity-mediated effects, where higher stomatal conductance facilitates water molecule dissociation, particularly pronounced in species with high transpiration rates like *Cinnamomum camphora*. These specific mechanisms collectively shape the observed spatial patterns of air ion concentrations across different sites (11, 21).

### 4.2 Effects of environmental factors on NAIs concentrations

The findings of this study reveal that relative humidity and temperature have a significant positive impact on NAIs concentrations, while air pressure and PM_10_ concentration exhibit a significant negative influence. Relative humidity emerged as one of the most significant positive predictors of NAIs concentrations. This finding is consistent with many studies highlighting the role of water vapor in the NAIs generation (9, 23). High humidity levels facilitate the formation of NAIs through processes such as the Lenard effect, where water droplet fragmentation releases negative ions (23). Water vapor can enhance the ionization of air molecules, further increasing NAIs concentrations (24). In urban green spaces, areas with higher humidity, such as those near water bodies or with dense vegetation, tend to exhibit higher NAIs levels (25). These findings underscore the importance of incorporating water features and moisture-retaining vegetation in urban green space design to maximize NAIs generation. Temperature also showed a significant positive correlation with NAIs concentrations, although this relationship is more complex and context dependent. Moderate temperatures are associated with increased plant activity, including photosynthesis and transpiration, which can enhance NAIs production (23). However, extremely high temperatures may reduce NAIs concentration due to increased evaporation and reduced humidity (25). In this study, the observed positive relationship suggests that the temperature range in the study area was conducive to NAIs generation. This highlights that careful consideration of local climatic conditions is crucial when designing urban green spaces to optimize NAIs benefits.

Air pressure has a significant negative impact on NAIs concentrations. High air pressure is often associated with stable atmospheric conditions, which can limit the vertical mixing of air and reduce the availability of ions (9). Conversely, low air pressure, typically associated with weather systems such as storms or frontal passages, can enhance NAIs production through increased atmospheric turbulence and ionization (9). These findings suggest that NAIs concentrations may vary significantly with weather patterns, emphasizing the need for long-term monitoring to capture temporal variations in NAIs levels. PM_10_ concentration exhibited a strong negative correlation with NAIs concentrations, consistent with previous studies (15). PM_10_ is positively charged and stays in the air for a long time, where it can combine with negatively charged NAIs, in turn changing its physical properties, forming macromolecular precipitates, and thus reducing the NAIs concentration (12). Urban areas with high traffic density or industrial activity are prone to elevated PM_10_ levels, which can significantly reduce NAIs concentrations in nearby green spaces (4). This emphasized the importance of reducing air pollution and implementing green infrastructure strategies, such as vegetation barriers, to mitigate the impact of PM_10_ on NAIs levels. In addition, Study suggest that NAIs concentrations during precipitation events compared to non-rainy conditions (26). This phenomenon can be attributed to rainwater-induced increases in surface humidity, which stabilizes NAIs by suppressing ion recombination rates (26). Consequently, strategic integration of aquatic elements (e.g., rain gardens, constructed wetlands) into urban design could amplify NAIs production, offering a scalable approach to enhance airborne ion-mediated ecosystem services in climate-vulnerable regions.

### 4.3 Implications for urban green space design

The observed relationships between environmental factors and NAIs concentrations have important implications for urban green space design and management. To maximize the NAIs generation, urban planners should prioritize the following strategies:

1. Incorporating water features: the positive influence of relative humidity suggests integrating water bodies, such as ponds, fountains, and streams, into urban green spaces to enhance NAIs production.
2. Optimizing vegetation density and diversity: high vegetation density and diversity can improve humidity levels while creating microclimates conducive to NAIs generation. Native tree species with high transpiration rates should be prioritized, since their enhanced water release capacity actively regulates local moisture dynamics. Furthermore, the configuration pattern of coniferous tree species plays a critical role, as their structural traits (e.g., needle-shaped leaves) are particularly conducive to increasing NAIs concentrations.
3. Mitigating air pollution: reducing PM_10_ levels through traffic management, industrial regulation, and green barriers can help maintain high NAIs concentrations in urban green spaces.
4. Adapting to local climatic conditions: the positive influence of temperature underscores the need to consider local climatic conditions when designing green spaces. In warmer climates, designers should implement strategies, such as shading and irrigation, to maintain moderate temperatures and humidity.

### 4.4 Limitation

The temporal aggregation approach involving fixed-interval averaging of NAIs measurements (morning, midday, and evening) may obscure critical diurnal fluctuation patterns, particularly missing transient peaks in ionization rates associated with dawn/dusk biological activity or anthropogenic emission cycles. Future investigations would benefit from high-resolution temporal sampling (e.g., hourly measurements) or continuous monitoring techniques to better resolve the circadian dynamics of NAIs and their environmental drivers. While this study identifies significant correlations between environmental factors and NAIs concentrations, it is important to acknowledge that these associations do not inherently confirm causality, as unmeasured confounding variables (e.g., vegetation type, wind patterns) or bidirectional interactions may influence the observed relationships. To disentangle direct causal mechanisms, future studies should prioritize controlled chamber experiments that systematically manipulate variables such as humidity and PM_10_ levels under isolated conditions, thereby isolating their specific effects on NAIs generation and dispersion.

## 5 Conclusion

Field monitoring across 240 sites revealed changes in NAIs concentrations among different urban green spaces and their influencing factors. With this study, we show that suburban forests consistently exhibit the highest NAIs levels, while roadside greenery, characterized by proximity to traffic-related pollution and limited vegetation density, shows the lowest concentrations. Analysis of environmental factors reveals that relative humidity and temperature positively influence the NAIs generation, while air pressure and PM_10_ concentration have a negative impact. These findings underscore the importance of integrating water features, optimizing vegetation density and diversity, and mitigating air pollution in urban green space design to maximize NAIs production and its associated health benefits. Future research should focus on long-term monitoring and the development of predictive models to further explain the mechanisms driving NAIs variability and support the creation of healthier urban environments.

## Data Availability

The original contributions presented in the study are included in the article/Supplementary material, further inquiries can be directed to the corresponding author.
